# Emerging Development of Auto-Charging Sensors for Respiration Monitoring

**DOI:** 10.1155/2022/7098989

**Published:** 2022-08-29

**Authors:** Hamza Abu Owida, Muhammad Al-Ayyad, Jamal I. Al-Nabulsi

**Affiliations:** Medical Engineering Department, Faculty of Engineering, Al-Ahliyya Amman University, Amman 19328, Jordan

## Abstract

In recent years, the development of biomedical monitoring systems, including respiration monitoring systems, has been accelerated. Wearable and implantable medical devices are becoming increasingly important in the diagnosis and management of disease and illness. Respiration can be monitored using a variety of biosensors and systems. Auto-charged sensors have a number of advantages, including low cost, ease of preparation, design flexibility, and a wide range of applications. It is possible to use the auto-charged sensors to directly convert mechanical energy from the airflow into electricity. The ability to monitor and diagnose one's own health is a major goal of auto-charged sensors and systems. Respiratory disease model output signals have not been thoroughly investigated and clearly understood. As a result, figuring out their exact interrelationship is a difficult and important research question. This review summarized recent developments in auto-charged respiratory sensors and systems in terms of their device principle, output property, detecting index, and so on. Researchers with an interest in auto-charged sensors can use the information presented here to better understand the difficulties and opportunities that lie ahead.

## 1. Introduction

Respiration is an important crucial indicator for humans because it can reflect trends in health conditions as well as the preponderance of respiratory diseases [1]. Abnormal respiration rate is also one of the indications of the severity of a COVID-19 patient's condition, and it is often used as an index to support clinical decisions for timely interventions at the inchoate stage of the infection. The SARS-CoV-2 viral infection could cause the severe acute respiratory syndrome, disturbing regular breathing and leading to continuous coughing [2].

Respiration involves a complex biological interaction between the central nervous system, respiratory-related motor neurons, and respiration muscles [3]. Based on these interactions, various techniques have been developed for respiratory rate detection. However, accurate, continuous, and pervasive respiratory rate measurement is challenging and still under investigation, especially for direct measurement [4].

Respiratory rate is commonly defined clinically as the times of respiration measured during a minute. Respiratory rate is a crucial indicator whose abnormality indicates major clinical occurrences. Measuring respiratory rate could reveal any disturbance in the bodily system that produces hypoxaemia [5]. Significant data suggests that an irregular respiratory rate is a predictor of potentially catastrophic clinical outcomes such as intensive care unit readmission, cardiopulmonary arrest, chronic heart failure, pneumonia, and pulmonary embolism [4].

Clinically, the respiratory rate could be measured using a variety of techniques, including spirometry, capnometry, and pneumography [4], as shown in Table 1. These procedures frequently necessitate complex and expensive gear that may interfere with natural breathing and can be unmanageable in specific applications such as ambulatory monitoring, stress testing, and sleep research. Automatic, dependable, and convenient sensors and equipment might significantly enhance the status of respiratory rate monitoring [5].

Despite this, respiratory rate is one of the most overlooked, under-utilized, and under-recorded vital signs. There are several causes for this; due to intense workloads and other issues, nurses rarely have time to complete a full 60-second measurement by manual counts. Inaccuracies occur when 2 or 4 multiply a 30-second or 15-second assessment [1, 5].

Interruptions, changing patients, difficulties counting or remembering a count, and poor visualization of the start and finish of a breath can all lead to further errors. Such problems connected with human counting can be alleviated by technologies that automate respiratory rate measurement. Medical device development has gained power in recent years as a result of technological advancements and increased lifespan. Electronic monitors might automatically measure Respiratory rate, which would have numerous advantages in clinical and physiological applications. Automatic respiratory rate monitoring has the potential to predict potentially significant clinical outcomes such as cardiac arrest or intensive care unit admission [4, 5].

Commercial and respiratory sensing systems have various drawbacks, such as being huge, costly, as well as complicated, thus limiting their use on a regular basis. The rational and practical research material is how to assess breathing limits in a continuous, straightforward, and comfortable manner. Researchers have devised a number of strategies to increase the comfort and feasibility of breath monitoring, including researching touch monitoring techniques, employing soft cloths, and more [6–8]. Experts in the field of biomedical sensing have taken a keen interest in autonomous respiratory nerves in particular [9–11]. Respiration is one of the most essential human characteristics; in order to detect respiratory disorders such as asthma, apnea, pneumonia, and tuberculosis, sputum samples must be collected and tested [12–16]. A significant role has been played by the most recent wearable and artificial sensors in the measurement of respiratory parameters, which include breathing rate and energy, temperature, and specific inert gases in exhaust air [17–22]. Both active and inactive nerves demonstrate unique gains in respiratory awareness among them [23, 24].

Triboelectric nanogenerators, piezoelectric nanogenerators (PENG), pyroelectric nanogenerators, hydroelectric nanogenerators, and hygroelectric nanogenerator devices are examples of artificial devices with biomechanical or thermal energy that can be converted directly into electricity [25–28].

The electrical impulses that they emit have a strong link to the respiratory tract. The airflow is driven by the TENG and can generate pulses that are correlated to the dynamics of flow of air [29, 30]. Since the demonstration of self-contained polyvinylidene fluoride respiratory sensors in 1990 [31], many advancements in material science and micro/nanofabrication technologies have been made [32–34].

Nanogenerators based on piezoelectric and triboelectric effects, in particular, encourage smaller, simpler, more sensitive, and precise autonomic respiratory and sensory systems that are more suitable for wearable and wearable circumstances [35–38]. Scientists have progressed to a detailed examination of nanogenerators utilized in bioengineering, with a special focus on TENG's future application and predilection for biological sensors such as respiratory monitoring [39–42]. Tai et al. provide an update on the emergence of respiratory analysis, which began with the possibility of moisture and gas sensitivity [43].

Furthermore, Su et al. recently published a study on TENG-enabled autonomous respiratory monitoring, which clearly demonstrates the sensitivity and model of your theater in both respiratory signal collecting and cellular respiratory analysis [44]. Due to its features including diminutive, low toxicity, light construction, simple make-up, and extensive applications, the nanogenerator-based respirator active biosensing is a critical component of the research and development of these systems. These self-regulating respiratory systems may also be used to connect smart remote monitoring sensors that become more common in the near future. From device-type features to output performance to respiratory sensor index, we have looked at the evolution of self-regulating respirator monitoring systems and systems. The air that is breathed through the mouth can stimulate self-regulating nerves, indicating direct breathing circumstances. Researchers have also created self-propelled heat sensors that use piezoelectric ceramic materials and nanogenerators can be used to trace gases in the lungs. In the meantime, we give problems and suggestions for breathing self-monitoring. Despite many notable improvements, several challenges remain, such as the difficulty of giving appropriate diagnostic information based on the effects of the autonomic nerves to date. However, artificial intelligence and other emerging, novel approaches to treating breathing impairments are being considered; we may be able to gather additional information on the impacts of the equipment they operate.

## 2. Application of Auto-Charged Sensors in Respiration Tracking

**Figure 1:**
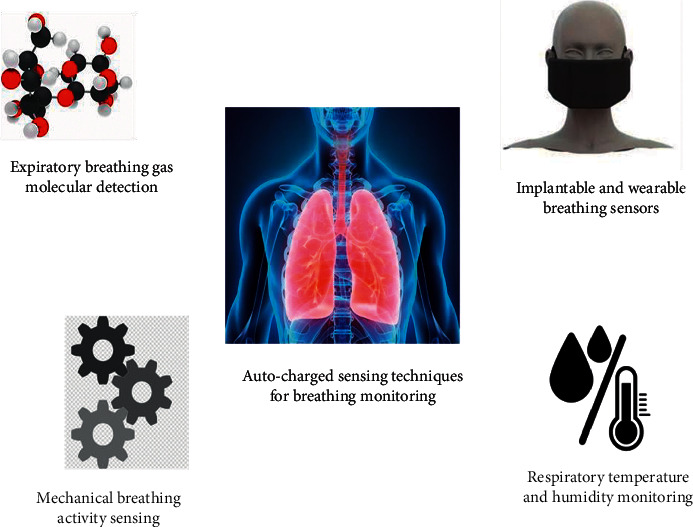
Auto-charged sensors application in respiration monitoring.

The concept of auto-charged sensors exhausting polyvinylidene fluoride or polyvinylidene difluoride strip in cantilever activation as a hearing aid was initially proposed by Roopa et al. in 2011 [6]. For a long time, the respiratory monitoring system's conscious stage was largely dependent on sensing applications with limited functionality and huge dimensions. Soon later, the usage of an autonomic respiratory monitoring system was created. They are separated into three functions: the collection of respiratory signals, the monitoring of respiratory temperature and humidity, and the detection of exhaust gas cells [45, 46]. They are divided into categories based on how they are used, with implanted and wearable ventilation systems being the most common as shown in Figure 1.

## 3. Respiratory Temperature/Humidity Monitoring

**Figure 2:**
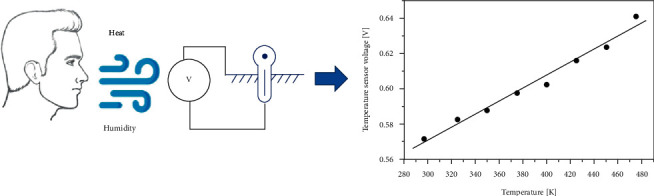
A schematic illustration of the respiratory temperature/humidity monitoring system.

Human respiration is hot and humid without mechanical energy [47–49]. Some self-regulating breathing systems detect respiratory temperature/humidity by gathering these symptoms (Figure 2). Xue and colleagues create a portable respirator pyroelectric sensing element with a component of polyvinylidene fluoride or polyvinylidene difluoride that could be activated thru breathing and has a high sensitivity to various respiratory conditions [50]. Due to its positive linearity and room temperature, the pyroelectric respirator sensor can also be used to detect ambient temperature. Roy et al. built the most flexible pyroelectric nanogenerator based on graphene in 2019 [51]. 4.3 V/kPa is possible. It also has a pyroelectric output power more than 1.2 nW/m2 with the degree of heat induced by continuous human breathing, indicating the potential for a pyroelectric respiratory sensor. Most of the self-activating respiratory sensors are dependent on the hydroelectric effect of graphene oxide, decreased graphene oxide, and so on [52]. In 2015, Zhao et al. first propose an auto-charged moisture sensing element to monitor breathing, which detects moisture signals based on single graphene oxide [53]. The graphene oxide mechanism that produces electricity from moisture is called the hydroelectric effect later [54, 55]. Mechanical ventilation is used to prepare a single graphene oxide, which can form a gradient of a group containing oxygen. When graphene oxide contacts with moisture, a concentrated H^+^ level can attract the movement of strong and free electrons in the outer cycle. Because of extreme sensitivity to changes in humidity, which have developed a self-regulating respiratory system to yield respiratory power, outgoing and current voltage can reach 18 mV, through the dampening effect of a normal male study.

After a range of exercises, graphene oxide can monitor the respiratory frequency and heart rate in a healthy patient. Following that, their team presented a stable dynamic transition based on the encounters of graphene oxide nanoribbon networks [46]. In the long run, there is a low probability of failure; the flexible effect membrane achieved high breathability for writing and reading. They also developed a breathable investigator battery using graphene oxide and lithium foil [56]. The graphene oxide film collects and transfers moisture in this battery, whereas the lithium foil reacts to redox moisture exposure. This work demonstrates promising power in the use of future respiratory biomedical sensor systems. Other researchers created self-contained artificial respiratory sensors based on the hydroelectric effect of graphene oxide [57–61]. Bo-skovic et al. introduced a humidity sensor based on an aluminum breathing monitor; no external power supply is required because of the low volume [62]. Using 1% silicone thin foam film as a composite capacitor, the air humidity sensor has the same functionality as an aluminum air battery. A structure with holes and nanochannels promotes the advertising and molecules of water distribution. To monitor changes in human skin moisture and respiratory rate, this sensor can be used.

## 4. Expiratory Breathing Gas Molecular Detection

**Figure 3:**
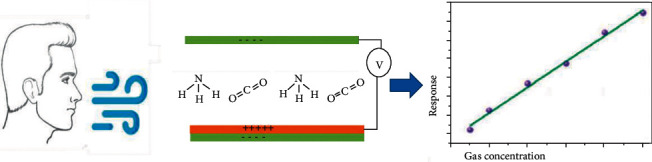
A schematic illustration of the respiratory exhaled gas monitoring system.

Efficient ventilators have detected some of the respiratory tract's gas emissions, providing a possible way to improve the structure of respiratory observing schemes and reinforce the connections between respiratory symptoms and disease identification (Figure 3). Using carbon dioxide concentration, odors in exhaust systems can be removed [7]. Kim and coauthors proposed a three-dimensional triboelectric respirator sensing element that is cast off to detect real-time respiratory signals, which are made up of polyethylenimine-coated ethylene propylene membrane with a willow coating [45]. This is one of the most significant mechanisms in the body for exchanging materials, such as humidity, temperature, and gas molecules, in between the body system and the environmental elements. The presence of nitrogen oxides, for example, is indicative of respiratory inflammatory diseases [63]; acetone is linked to diabetes [64]; and ammonia is linked to hepatitis [65–68]. Furthermore, alcohol-filled gas has been recognized as a sign of fatty liver and plays a key role in diagnosing a drunk driver [69, 70]. Investigating the voltage at the output, four breathing patterns can be detected by them, ranging from very strong to very weak to long and short. The power output of the solid mode is almost three times that of the weak one, while the short and long breathing case has the same discharge pattern of 0.5 seconds with a maximum value variance. By inventing new inventions, they use polyethylenimine as a CO_2_ scanner to differentiate between expiration and respiration due to differences in CO_2_ concentration. The triboelectric respiration sensor emissions decreased by 11.73% after CO_2_ was injected at a speed of 6.5 m/s for an extended time period with a relative humidity of 37%.

The amount of alcohol in the exhaust gas is a good predictor of safety and can be used to test for drunk driving. Derived on a triboelectric nanogenerator that is driven by a blow, Wen et al. developed a self-regulating alcohol respirator [71]. Triboelectric respiration sensor identified CO_2_ sensitivity in human respiratory monitoring and fostered the evolution of self-regulating respiratory neurons. It features sensors that range from 10 to 200 ppm and a response/recovery time of 11 to 20 seconds. Xue et al. establish that alcohol-filled breathing is beneficial to human respiration [72]. Triboelectric effect, gas sensitivity sandwich nanostructures, and elastin are merged in this e-skin. When an adult hits it, this self-driving system can detect an intoxicated driver. Their team also established respiratory sensors using piezo-gas-sensing arrays made of polyvinylidene fluoride. As a result, five different senses elements have identified responses that correspond to specific gas signals [73].

Therefore, this device has been proven to be useful in detecting ethanol saturation in the exhaust system. All of these activities encouraged the systematization of self-regulation and greatly improved their performance. An increase in ammonia in the concentration of exhaust gas indicates a certain disease. Nanocomposite films for respiratory strength and trace concentrations of NH_3_ have been developed by Wang et al. in 2019 [74]. Subsequently, they developed a triboelectric self-powered respiratory sensor and adopted a theoretical model for human respiratory analysis and the function of the NH_3_ sensor [75]. However, NH_3_ sensor is important to the triboelectric respiration sensor. If connected to the chest through an external gas collection system, the triboelectric respiration sensor can collect energy and rhythm of breathing from normal expansion and contraction of the chest, recognizing the monitoring of gas emissions. Triboelectric respiration sensor output function refers to triboelectric respiration NH3 biomarker traces in outdoor electricity that can be monitored with this technology, and the different types of breathing in the zip hatha tradition (standard, deep, shallow, and rapid) are distinguished as is the standard procedure. Nitrogen dioxide, a harmful gas, is found all around us. TENG was used by Su et al. to develop a portable alveolus-inspired membrane sensor for monitoring human respiration and detecting NO_2_ [76].

Alveolus-inspired membrane sensor emissions can show differences in time and respiratory power. This function provides a new way to sense toxic gases by merging breathing observing and NO_2_ recognition. Internally and externally targeted gas may cause a potential electrical gap between the latex membrane and the sensitive film by expanding and contracting. Expending a composite film of tungsten trioxide to detect gas sensation, the Alveolus-inspired membrane sensor revealed admirable sensitivity of under a concentration of 80 ppm of NO_2_, up to 340.24%. Compared to other gases, the Alveolus-inspired membrane sensor also exhibits a higher NO_2_ sensor choice.

## 5. Sensing of Mechanical Breathing Activity

**Figure 4:**
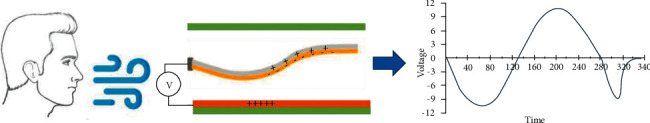
A schematic illustration of the respiratory mechanical monitoring system.

A number of mechanical indicators, such as the compression of the sternum and the flow of air throughout inspiration and expiration (Figure 4), are reversed by our respiratory system [77, 78]. Many autonomous respiratory systems, on the other hand, rely on pressure impulses to monitor changes in the physical state of an object mechanically [79–83]. In recent years, size and output features in diverse sensory perceptions have been used to more accurately portray self-regulating respiratory system systems for the gathering of respiratory tract data. The position of the respiratory tract has a significant impact on the signalling system, creating a shift in the location of the machines that determine whether they are driven by body movement or airflow. Body motions are the primary source of power for the nerves implanted in the belly, chest, and throat.

They frequently have a larger output than other air-driven airflow due to their high mobility. High temperatures, humidity, and the detection of molecules, on the other hand, are reflected in the gas-collecting respiratory systems in the nose and mouth. Breathing sensors are frequently implanted to collect energy and detect mechanical signals and the mouth. Breathing sensors are frequently implanted to collect output breathing mechanical activity. Predicated on the observation of direct conversions of communication stress, one study recently launched a bilayer triboelectric sensor for sensing pressure, with resolutions of 0.34 Pa and 0.16 Pa related to the variations in the level of air pressure [84]. There may be sporadic interaction between one of the rubber films and the fluorinated ethylene propylene membrane due to movement, breathing, and heart rate, which could signal a shift in pressure. The technology can identify self-sustaining breathing monitoring in real time when coupled to an airbag and connected to the study center. Liu et al. created a human respiratory monitoring system that can feel pressure based on the microsphere-based TENG [85].

A thin triboelectric layer made up of thermal expanding microspheres and polydimethylsiloxane is the most critical component of this TENG; different points of interaction are created by changes in pressure and hence variable charges on the triboelectric surface. Sensitive to changes in breathing position, this 33 mm^2^ pressure gauge can provide consistent output signals when connected to the chest of the head. At 30 breaths per minute and 9 breaths per minute, the recorded signal revealed a substantial difference between shallow and deep breaths, respectively. Furthermore, the microsphere-based sensor may be utilized to detect hand signals, which can help with noninvasive medical diagnostics by presenting key symptoms.

The majority of respiratory signal monitoring devices rely on direct contact between electrical objects and individuals [86–88]. Chen et al., on the other hand, saw a gap in the research of unaffected respiratory nerves and developed a microstructure sensor with an auto-charged gauge sensor that detected data gathering without direct skin contact [89]. The electrostatic events that occur between the highly charged multilayered elements are the primary mechanism of action. Auto-charged microstructure sensor can screen sensitive breathing and heart rate in a noninvasive mode while operating under body weight, consistent with significant differences between the multiple processes of a wholesome subject. The advance of offline auto-charged microstructure sensors has enhanced the sense of well-being of users, which has encouraged the review of monitoring systems for the health of people's sleep patterns. Furthermore, researchers have introduced a breathing apparatus that works by directly touching the skin. The majority of these are entirely predicated on the sensor that builds up near the chest, abdomen, and throat. As a result of the skin's proximity to the breathing nerve within those locations, the breathing signals can be directly obtained from the skin. Generally, the acquisition of mechanical breathing activity technology is easier than inhaling and exhaling through the nose and mouth because body movements are greater than the force of air currents. In addition, energy-efficient respiratory nerves can also be driven by airflow produced by respiratory behaviour. Wang et al. introduced a TENG respirator-driven air sensor, in which a thin film of nanostructured polytetrafluoroethylene flexible was usually vibrated with an exposed to airflow; acrylic nozzle becomes transparent [30]. TENG has a corresponding reaction to different respiratory conditions, and the increasing amount of charge passed through the respiratory process is closely related to the total amount of gas converted.

## 6. Implantable and Wearable Breathing Sensing Elements

**Figure 5:**
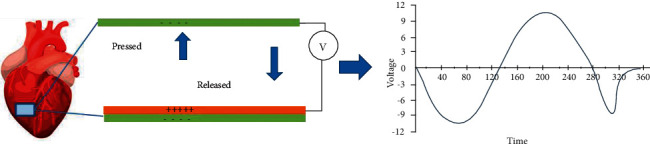
A schematic illustration of the respiratory implantable monitoring system.

Considering the issue of safety and ethical testing, a few studies on the collection of breathing signs were performed *in vivo.* Animal studies have shown that a few related studies can be carried out. The heart, lungs, and diaphragm can harvest energy from rest and relaxation as integrated biomedical systems (Figure 5), allowing people to breathe naturally for the first time [90]. Using an ultrastretchable micrograting system, Li et al. designed nanogenerator prostheses capable of powering implanted medical devices [20]. After being implanted into the abdominal cavity of Sprague-Dawley adult rats, it can harvest energy from the regular diaphragm movement during respiratory process and output electrical signals correlated with the inhale and exhale part. Nowadays, portable electronic surveillance devices are often self-powered, configured, and operated in a variety of ways [91, 92].

As living standards increase and the level of education increases, autonomous respiratory systems have a much lower tendency for performance, ease, and efficiency [93–95], addressing social issues through the creation of artificial and wearable nerves [96–99]. The aforementioned self-regulating breathing apparatus is an electronic device that is worn extensively, facial or abdominal masks, or any combination of these items. But implanted sensors have many advantages, such as high accuracy and the ability to monitor specific biomedical signals for an extended period of time [92, 100]. However, the development of implanted monitoring devices is less battery life [101]. An energy-efficient respirator system usually combines energy harvesting work with the collection of respiratory symptoms. Ma et al. established an effective triboelectric sensor input for monitoring the human body in real time [102]. It is made up of triboelectric layers, electrodes, and spacers, and all structures are closed with a flexible multilayer shell. Packed in a pericardial sac, the implantable triboelectric active sensor not only can monitor respiratory rhythm and blood pressure. During the breathing process, the implantable triboelectric active sensor output peaks rose from 4.8 V to 6.3 V when breathing and descended to the real state of ventilation. Zheng et al. designed a multimodal triboelectric nanogenerator as a self-regulatory health monitoring system [103].

When powered by a Yorkshire pig heartbeat, it can generate an open-circuit voltage of 14 V and a short-circuit current of 5 *μ*A. In addition, the implantable triboelectric nanogenerator has output peaks that correspond to the respiratory cycle. Moreover, it was introduced the function of implantable triboelectric nanogenerator using the harvesting force of respiratory mechanical action [92]. Following that, inserted under the chest of the left mouse, it can measure the vital volume of the forced mouse. Implantable triboelectric nanogenerator demonstrates the potential to develop health facilities that incorporate artificial intelligence. Compared to artificial energy sensors, wearable sensors are more flexible and easier to replicate [10, 93, 104, 105]. The wearable respiratory sensor can be used in conjunction with a variety of other wearable devices, including face masks [106] and chest/abdomen belts [107, 108]. Using electrospun polyetherimide nonwoven as electret materials, Cheng et al. produce an energy-efficient wearable face mask [109]. A smart face mask can eliminate submicron particulate matter more efficiently and harvest energy from airflow based on the cost that retains polyetherimide potential during severe humidity conditions. The mask can be used as a self-powered biomedical sensor to detect breathing levels during human respiratory movements. A normal person breathes roughly 21 times each minute. The effectiveness of a filter can be measured using an LCD display and this technique. Respiration is monitored via graphene fibers that use moisture-electric energy transformation to collect energy from the environment [110].

This gadget can detect changes between different human breathing conditions when coupled to a mask and has a maximum output voltage of 292 mV. Advanced diatom frustrations of cellulose nanofibril-based TENG respiratory monitoring were introduced by Rajabi-Abhari et al., capable of producing an average of 85.5 mW/cm^3^ with an effective interaction capacity of 4.9 cm^2^ in isolation mode [111]. They have designed a smart mask that uses discarded energy because of this DF-CNF TENG. When a person breathes, airflow will cause the fluorinated ethylene propylene film to move between the two layers of the diatom frustules enhanced cellulose nanofibre, thus producing the energy to release energy associated with respiratory conditions. The single-TENG mode has a lower voltage than the TENG-based dual-sensing breathing sensor. Because of its small size and high output, this sensor will be useful in future skin-attached wearable health monitoring systems. Additional research is being done on flexible fabrics, e-skin, and chest/abdomen bands [112–114], as well as smart facial masks. Using a chest movement harvester, Shahhaidar et al. in 2013 developed a self-powered respiratory biosensor [115, 116].

They have created piezoelectric and electromagnetic sensor/harvester devices that can be added to the chest band. In 2015, they created a new wearable electromagnetic self-powered sensor that was also attached to the chest band [117]. It is capable of keeping track of the breathing process by gathering the power produced by the movement of the chest wall by implanting this neural system in the xyphoid system and the navel. Vasandani et al. develop a portable respirator with a microdome pattern (wREH) based on contact separation mode [118]. The low-density PDMS' soft design aims to improve wREH output performance. To track breathing levels and depth, wREH can be attached to a belt that does not follow the rules imposed around the abdomen. The open-circuit voltage reaches 16.8 V under typical respiratory test conditions, revealing the real power of future biomedical sensory networks.

## 7. Conclusion and Perspectives

The growth of biomedical monitoring programs, particularly respiratory monitoring programs, has increased in recent years as a result of the growing promotion of healthy living and quality. In this comparison, the benefits and drawbacks of the most recent respiration monitoring systems by different types of signal collection are discussed with their perspectives. Flow variations, including the duty cycle, intensity, and density of cross ventilation throughout breathing, have a close association with the output of the respiratory tract driven by airflow. Although the pyroelectric and hydroelectric effects of the respiratory system are mainly dependent on the temperature and humidity of the exhaust gas, respectively, it is easily modified by external forces. In addition, the respiratory sensor for cell detection can provide extra information on the chemical composition, which can help with disease diagnosis. Furthermore, by combining the diverse qualities of the internet of things, an intelligent healthcare system has spurred the development of powerful artificial electronics. However, there are still some challenges that need to be overcome for the development in the future, and they are described as follows.

### 7.1. Miniaturization

The quick progress of energy-based respiratory monitoring studies is encouraging. Many of the incredible self-regulating breathing systems are based on a combination of respiratory symptoms such as nasal congestion, mouth breathing, and chest motions. Airflow, humidity, temperature, and molecules are all detectable in the exhaled gas, but chest motions provide primarily mechanical information in the form of pressure changes. The majority of respirators are built into masks, belts, or belts. Resources should be kept as few as possible to promote user comfort. Some researchers are now using flexible materials to produce energy-efficient breathing sensors, hence improving user comfort. External power dependencies can be eliminated with independent sensors, which have the benefit of lowering device ratings. Advanced micro-/nanofabrication technologies, on the other hand, may offer a more complex way to produce an integrated and compact system. Personal wearable equipment will be possible in the future because of a flexible breathing mechanism built of microscopic pledges.

### 7.2. Stability

Respiratory rhythm, respiratory capacity, acetone, respiratory moisture, and other physical and biochemical indicators of respiratory activity may reflect the health or sickness of the human body. If other devices can detect many of the above symptoms in real time and simultaneously, the devices' stability cannot be overlooked. Developing a system encapsulation method, such as wrapping a device in tiny layers of expandable polymers, could improve its stability. To acquire reliable signals, most respiratory monitoring equipment should be limited to a specific location during this time. The respiratory system's antijamming capabilities and functional health are critical for future development. Maintaining consistent sensory qualities of the energy system during daily life is a huge problem, especially in real time and for long-term acquisition.

### 7.3. Improved Output

The power supply limit still exists for the most recent self-operating sensors or devices. Although harmful methods such as piezoelectric, triboelectric, and pyroelectric devices can harvest energy from the environment, the energy efficiency of the entire respiratory monitoring system is insufficient to match the power consumption of the system. A major task will be to increase the performance of the harvesting power system in order to drive the system in real time. The sensor data for the self-regulatory respiratory system should then be delivered to terminals such as computers and mobile phones, which will interpret the data and provide an analysis of the users' respiratory health. Wireless data transmission is required to ensure the device's wearability. The devices' power consumption increases as a result of the wireless transmission of received signals. The average power consumption, for example, ranges from a few millimeters to tens of millimeters, which is higher than the output of several devices harvesting less wearable electricity.

Nanomaterial-based sensors have been developed by researchers to diagnose respiratory disorders. However, a reliable medical diagnosis is still a long way off. Another goal of the respiratory monitoring program is to give clients and physicians the findings of prior tests as well as professional recommendations. Worse, the most recent artificial respiratory nerves are unable to deliver appropriate results in terms of respiratory disease diagnosis. The fundamental obstacle is that each user's distinct breathing variances will generate signal differences, posing numerous challenges in accurately diagnosing respiratory disorders. One possible option is to employ artificial intelligence to deal with symptoms, which has been used extensively in disease diagnosis. Self-regulating respiratory systems for modern medical diagnostics and healthcare, on the other hand, should be multitasking.

A diagnosis of the respiratory disease typically necessitates the collection of health data on a variety of factors, including the amount of gas moved, respiratory frequency, and organic variables in the respiratory system. The rapid growth and popularity of 5G have recently brought considerable benefits to a smart city's wireless sensor system, indicating that it is a major artificial intelligence site with high accuracy for respiratory sickness. Hopefully, we will soon be able to benefit from the convenience of digital communication via wireless self-driving sensors.  Table 2 summarized, and contrasted the most recent auto-charging breathing monitoring systems in terms of their advantages and challenges.

## Figures and Tables

**Table 1:** Current mainstream methods used for clinical respiratory rate monitoring.

Methods	Advantages	Disadvantages
Spirometer	Accurate, simultaneous measurement of multiple respiratory parameters	Interfere with natural breathing, difficult for continuous respiratory rate monitoring
Capnometry	Accurate, easy to perform, able for continuous RR monitoring, simultaneous measurement of biochemical parameters	Uncomfortableness caused by contact, special devices needed for analysis
Manual counting	Easy to perform, noncontact	Inaccurate, time-consuming
Impedance pneumography	Accurate, continuous, simultaneous measurement of multiple respiratory parameters	Difficult to perform, special devices needed for analysis

**Table 2:** Recent auto-charged breathing monitoring systems are summarized and compared in terms of their advantages and challenges.

Signals	Advantages	Disadvantages	Perspectives
Molecular	High output voltage Low cost Highly accurate Detection of multiple target markers	Easily affected by the surrounding environment	Improving material quality, structural design, and encapsulation all work together to increase structural stability
Temperature/humidity	Simple structure Light weight	Slow response speed Easily affected by the surrounding environment Expensive	Modifying the material to speed up the response time Improving stability and broaden material options
Mechanical	High sensitivity Simple structure High output voltage Cost-effective	Susceptible to outside environment	Improving material quality, structural design, and encapsulation all work together to increase structural stability Miniaturization

## Data Availability

The data used to support the findings of this study are available from the corresponding author upon request.
